# Street-level workers’ inadequate knowledge and application of exemption policies in Burkina Faso jeopardize the achievement of universal health coverage: evidence from a cross-sectional survey

**DOI:** 10.1186/s12939-017-0717-5

**Published:** 2018-01-08

**Authors:** Valéry Ridde, Gerald Leppert, Hervé Hien, Paul Jacob Robyn, Manuela De Allegri

**Affiliations:** 1 CEPED, IRD, Université Paris Descartes, Inserm, équipe SAGESUD, 45, rue des Saints Pères, 75006 Paris, France; 2 IRD (French Institute For Research on Sustainable Development), CEPED (IRD-Université Paris Descartes), Universités Paris Sorbonne Cités, ERL INSERM SAGESUD, Paris, France; 3 German Institute for Development Evaluation (DEval), Fritz-Schäffer-Str. 26, 53113 Bonn, Germany; 4 Centre MURAZ, Bobo-Dioulasso, Burkina Faso; 5 Health, Nutrition and Population Global Practice, The World Bank, 701 18th St NW, Washington, DC 20006 USA; 6 Institute of Public Health, Medical Faculty, Heidelberg University, Im Neuenheimer Feld 130.3, 69120 Heidelberg, Germany

**Keywords:** Street-level workers, Public policy, Health policy, Implementation gap, Exemption, Free care, Universal health coverage, Burkina Faso

## Abstract

**Background:**

Street-level workers play a key role in public health policies in Africa, as they are often the ones to ensure their implementation. In Burkina Faso, the State formulated two different user-fee exemption policies for indigents, one for deliveries (2007), and one for primary healthcare (2009). The objective of this study was to measure and understand the determinants of street-level workers’ knowledge and application of these exemption measures.

**Methods:**

We used cross-sectional data collected between October 2013 and March 2014. The survey targeted 1521 health workers distributed in 498 first-line centres, 18 district hospitals, 5 regional hospitals, and 11 private or other facilities across 24 districts. We used four different random effects models to identify factors associated with knowledge and application of each of the above-mentioned exemption policies.

**Results:**

Only 9.2% of workers surveyed knew of the directive exempting the worst-off, and only 5% implemented it. Knowledge and application of the delivery exemption were higher, with 27% of all health workers being aware of the delivery exemption directive and 24.2% applying it. Mobile health workers were found to be consistently more likely to apply both exemptions. Health workers who were facility heads were significantly more likely to know about the indigent exemption for primary health care and to apply it. Health workers in districts with higher proportions of very poor people were significantly more likely to know about and apply the delivery exemption. Nearly 60% of respondents indicated either 5% or 10% as the percentage of people they would deem adequate to target for exemption.

**Conclusion:**

This quantitative study confirmed earlier qualitative results on the importance of training and informing health workers and monitoring the measures targeting equity, to ensure compliance with government directives. The local context (e.g., hierarchy, health system, interventions) and the ideas that street-level workers have about the policy instruments can influence their effective implementation. Methods for remunerating health workers and health centres also need to be adapted to ensure equity measures are applied to achieve universal healthcare.

**Electronic supplementary material:**

The online version of this article (10.1186/s12939-017-0717-5) contains supplementary material, which is available to authorized users.

## Background

While decision-makers are essential in ensuring the success of public policies, studies have shown the key role of street-level workers in implementation of public policy [1]. In fact, political will and the allocation of public funds for healthcare are not enough to ensure that a planned policy will become a reality [2]. Labeled as “policy implementers” or true “policy-makers”, street-level workers are frontline health workers who operate at the interface between decision-makers and the beneficiaries of policies. In their position, they can exert considerable influence on their “effective implementation” [3]. In the context of Burkina Faso, street-level workers are, for example, nurses, midwives, doctors and mobile health workers, who are in direct contact with health care users and who are asked to apply state-led user fees exemption measures for indigents. Street-level workers decide whether they ignore or implement these measures. Through their actions, their “dispositions” [3], their interpretations of the policy instruments (e.g. exemption), or even their ideas about those instruments, they can considerably influence a policy’s implementation [4, 5]. Moreover, while the number of quantitative studies on the implementation of public policies is growing, they are still less common than qualitative approaches, and very rare in Africa [6].

In the African Region, evidence on the implementation of public policies is still limited, both in the field of health [7, 8] and in the study of the role of street-level workers [9]. Yet we know that street-level workers’ coping strategies in Niger or Mali [10, 11] and their relationships with communities in Burkina Faso [12] have had strong impact on the implementation of recent policies to subsidize the costs of healthcare. Additionally, studies that assess the role of street-level workers in implementation of exemption policies targeting those who are socially and economically disadvantaged are even more rare [13].

On global level, user fees exemption policies have been recognized as essential measure for nations to move towards universal health coverage (UHC). Exemption policies are based on the recogniction that certain segments of the population are simply not able to pay for the care they need, not even in tax-based or social health insurance systems [14]. Hence, States are constantly confronted with the need to decide who should be exempted and how to fund such exemption mechanisms. The current global discourse on UHC points at the objective not to leave anyone behind [15], otherwise exemplified by a statement made by a relevant WHO consultative group pointing at an unacceptable implicit trade-off when decisions are made "to expand coverage for well-off groups before doing so for worse-off groups" [16].

A collective work published in French presented several analyses of these types of public policies in African countries [13]; however, these analyses were mainly qualitative. In Ghana, a study based on 13 interviews revealed the low level of knowledge among health professionals, as street-level workers, regarding exemption measures for persons under 18 years of age in the context of the national insurance system [17]. In Sudan, a qualitative study with 177 key informants showed that their levels of knowledge about the content of the user fee exemption policy varied greatly [18]. In South Africa, Niger, and Burkina Faso, three quantitative surveys used the same structured questionnaire to measure health workers’ perceptions regarding exemption policies, but the sample sizes were small and the analyses were descriptive [19–21]. These studies showed that health workers are relatively satisfied with the exemption principle, but are relatively unhappy with the implementation challenges of these policies. Health workers also worried about the sustainability of these policies and the ability of States to maintain them over time.

To our knowledge, no large-scale study in Africa has measured street-level workers’ level of knowledge and application of exemption measures. Yet “compliance with statutes’ directives” is one of the dimensions of “successful implementation” [3]. As such, it is important to gather evidence on this subject, since we know that, in Burkina Faso, exemption measures targeting indigents for primary healthcare that date back to 1993 have not yet been implemented [22] and those of 2007 targeting indigent pregnant women at deliveries are implemented very sparsely [23].

Thus, the primary objective of this study was to measure and understand the factors associated with health workers’ knowledge and application of user fee exemption policies targeting primary healthcare for indigents and deliveries for indigent women. Both policies are meant as part of a wider strategy to promote UHC in Burkina Faso. The secondary objective was to understand their preferences regarding what proportion of the population might appropriately be considered indigent in order to qualify for this payment exemption.

## Methods

### Hypotheses

Given the very low use of health services by indigents in Burkina Faso [24], we hypothesized that street-level workers’ knowledge and application of exemption measures were also very low. In addition, given the pyramidal structure of the health system, we hypothesized that street-level workers’ knowledge and application of these measures would increase in proportion to their levels of education and job responsibility. However, we also expected that personnel working in obstetric services would be more knowledgeable about the user fee exemption for indigent women’s deliveries and would apply those measures more often than would others. Given the conflict of interest that arises because health workers receive, from the facility management, part of the fees paid locally by users for curative consultations [25], we hypothesized that the application of the general exemption would be lower than that of the delivery exemption, which is reimbursed by the central level.

### Study setting

Burkina Faso is a French-speaking landlocked country in West Africa. It has a population of approximately 18 million people, mostly engaged in the informal sector, primarily in subsistence agriculture [26]. With an annual per capita Gross Domestic Product (GDP) of USD 650 (2016), Burkina Faso is one of the poorest countries in the world, where nearly 44% (2014) of the population live below the national poverty line of USD 1.90 a day [26].

In 2014, total health expenditure in Burkina Faso amounted to nearly 5% of GDP, with private out-of-pocket expenditure accounting for nearly half (48%) of this amount. Health service provision is organized in a three-tier system, with first-line facilities (Centre de Santé et Promotion Sociale – CSPS) located in the rural areas, second-line facilities in the district capitals, and tertiary referral facilities located in the regional capitals and in Ouagadougou. Health service provision continues to be characterized by large inequalities between rural and urban areas, mostly due to problems linked to inadequately equipped and staffed health facilities [27].

In the early 1990s, the Bamako Initiative, combined with the introduction of structural adjustment policies, resulted in user fees being introduced at all levels of the health sector [28]. Since then, similarly to what has been observed elsewhere, user fees in Burkina Faso have been proven to impede access to care and to expose households to catastrophic health expenditure [29].

In an attempt to enhance equity in access and foster financial protection in a system largely based on user fees, the government of Burkina Faso has enacted two measures in the years since the 1993 Bamako Initiative. First, in January 2007, when passing legislation to reduce user fees for obstetric services, the government decided to exempt the poorest quintile of women from paying any fee, including the reduced co-payment for delivery services foreseen by the new policy. This 2007 measure was funded by the State’s national budget from 2007 to 2015 [30]. Second, starting in 2009, the government prescribed that the worst-off (*les indigents*) should be fully exempted from paying user fees for all preventive and curative services provided by public facilities. Local health management committees were mandated to fund these exemptions by redistributing resources acquired via cost-recovery schemes. Throughout their implementation, neither measure has systematically included any specific effort to identify potential beneficiaries, leaving it to individual providers to implement the exemption.

These two exemption measures are the focus of our analysis.

### Study design, data sources, and sampling

To assess knowledge and application of the two exemption measures described above, we used cross-sectional data from the baseline round (carried out between October 2013 and March 2014) of the health worker survey conducted as part of the impact evaluation of a performance-based financing (PBF) intervention [31].

In line with the World Bank toolkit for impact evaluation [32], the structured survey collected information on individual health workers, specifically: age, sex, and years on duty; income and its sources; training and clinical knowledge; working conditions; and motivation and satisfaction with multiple aspects of daily work. Given our specific research interest in exemption policies, we added to the standard survey tool a section specifically exploring their knowledge and implementation of existing policies and their attitudes towards forthcoming ones.

The survey targeted 1521 health workers in all 498 first-line (CSPS), 23 second- and third-line (district and regional hospitals) facilities, and 11 private or other facilities included in the sample and distributed across 24 districts. The sample included a census of all public and private facilities in 12 intervention districts and a random sample of one-third of all public and private facilities in control districts. Research assistants were instructed to interview as many health workers as possible during their one-day stay at each facility. In practice, this resulted in approximately three health workers surveyed per facility, which is equivalent to 80% of all health workers present on facility premises on the day of the survey (the remaining 20% not being interviewed due to unavailability or lack of time, not due systematic screening by the interviewers or to an explicit refusal). Our sample included an ample variety of health workers: doctors, registered nurses, assistant nurses, midwives and assistant midwives, as well as mobile health workers (*agents itinérants de santé, AIS*). We purposely administered the survey to this wide range of health workers and kept them all in our analysis because they all interacted with patients to provide care and, as such, all made decisions on the application of exemption policies [27].

We did not restrict our analysis to a specific cadre, as in other analyses based on the same survey [33]. However, due to missing data, the effective sample varied across outcome variables. Specifically, we could obtain complete information for: a. between 1345 and 1314 health workers on the primary outcomes (for indigent exemption and delivery exemption respectively); b. between 1503 and 1472 health workers for the secondary outcome. Descriptive statistics on 1311 health workers, due to missing values in the explanatory variables.

### Variables and their measurement

Table 1 provides an overview of all outcome and explanatory variables included in the analysis and their measurement. Specifically, we defined four primary outcome variables: two to assess knowledge of the two above-mentioned exemption measures and two to assess the application of the two measures. All outcome variables were measured using a dichotomous scale.

**Table 1:** List of variables and their measurements

Variables	Measurement
Outcome indicators	
Knowledge of directives regarding exemptions for indigents	0 = No; 1 = Yes
Application of indigent exemptions in facility (recoded) *Note: Question was recoded to 0 if response to filter question “knowledge of directive for indigents” was “no”*	0 = No; 1 = Yes
Knowledge of directive regarding delivery exemptions for indigents	0 = No; 1 = Yes
Application of delivery exemptions for indigents (recoded) *Note: Question was recoded to 0 if response to filter question “knowledge of delivery exemption directive” was “no”*	0 = No; 1 = Yes
Individual characteristics	
Sex of health worker	0 = Male; 1 = Female
Age of health worker *Note: Variable was centred for regression models*	Continuous
Education of health worker (ordinal)	0 = No formal education or primary 1 = Junior high school 2 = Senior high school 3 = Higher education
Health worker type	1 = Nurse (IDE, IB, nursing assistant) or medical doctor 2 = Midwife (midwife/birth attendant/assistant midwife (AA, AB)) 3 = Mobile health worker (AIS)
Head of health facility	0 = No; 1 = Yes
Years worked at facility *Note: Variable was centred for regression models*	Continuous
Days of absence in past 30 days *Note: Variable was centred for regression models*	Continuous (0–30)
Performance feedback received during past 12 months *Note: 453 cases imputed due to missing values*	0 = No; 1 = Yes
Training in emergency obstetric and neonatal care	0 = No; 1 = Yes
Training – pro-poor targeting	0 = No; 1 = Yes
Training – user fee exemption	0 = No; 1 = Yes
Training – health insurance	0 = No; 1 = Yes
Training – facility (financial) management	0 = No; 1 = Yes
Influence on decisions in facility (perception)	Scale: 0 = lowest to 10 = highest level
Control over facility (perception)	Scale: 0 = lowest to 10 = highest level
Facility-level variables	
Urban/rural	0 = Rural 1 = Urban
Health facility level	1 = Primary 2 = Secondary 3 = Private
Number of clinical staff at facility	Continuous
District-level variables	
Indigent household share in district (20th percentile of asset index) *Note: household-level variables aggregated on district level*	Continuous (0–1)
Secondary outcome indicator	
Preference for selection of x% of the population to be fully exempted	Categorical variable (1%, 5%, 10%, 20%, 25%)

Knowledge and application for exemption measures for preventive and curative services were initially assessed with the questions outlined below, whereby we later categorized yes as 1 (one) and no as 0 (zero):

- The planning guidelines of the Directorate of Studies and Planning since 2009 require that each health center uses 200,000 F CFA per year for the user fees exemption of indigents. Question text: Are you aware of this directive? (Response: yes or no)
- Question text: Have you applied it in your health center? (Response: yes or no)

Knowledge and application for exemption measures for for delivery services were initially assessed with the questions outlined below, whereby we later categorized yes as 1 (one) and no as 0 (zero):

- As part of the national subsidy for deliveries since 2007, it is expected that 20% of pregnant women are considered indigents and therefore exempt from payment of 900 F CFA. Question text: Are you aware of this directive? (Reponse: yes or no)
- Question text: Have you applied it in your health center? (Response: yes or no)

We purposely assessed knowledge and application at the level of the individual health worker and not at the facility level because, as mentioned, the government left the responsibility for identifying potentially eligible individuals up to individual health workers, with no specific role in this process assigned to the facility administrative manager. Thus, based on our experience in the country, we expected to observe variation in both knowledge and application, even within individual facilities.

Most explanatory variables are self-evident and reflect the health workers’ sociodemographic profile. Education is listed as an explanatory variable because, as mentioned, our sample included lower-level workers, such as the AISs. Thus, we expected variation in education levels (Additional file 1). To counteract problems due to small samples within single occupational types, we grouped health workers into four major categories: medical doctor (very few observations) or nurse; midwife; assistant midwife; and AIS [27]. The variable “dual practice” assessed whether a health worker held an additional permanent post. The variable “performance evaluation in the last year” measured whether the health worker had undergone an evaluation within the previous year. Six dichotomous variables assessed whether the health worker had received training in any of five specific fields over the past 12 months (emergency obstetric care and neonatal care; pro-poor targeting; user fee exemption; health insurance; facility financial management). Two variables measured health workers’ perceptions regarding their capacity to influence decision-making processes and control work at their facility. Both variables elicited answers on a Likert scale with values ranging from 0 to 10.

Five variables measured facility-level characteristics, specifically: rural vs. urban location; facility type (1 = primary facility; 2 = secondary facility – including the few regional hospitals included in the sample; 3 = private); district; number of clinical staff assigned as personnel; and proportion of very poor people residing in a specific district. This last variable was computed using data from the parallel household survey conducted in the catchment areas of all facilities included in our sample. We computed this variable by looking at how many people were listed in the lowest wealth quintile in each of the districts. To generate this variable, we first computed an asset-based wealth index for the overall sample (i.e., pooling data from all 24 districts) and then checked what proportion of very poor resided in each district. We treated this as a continuous district-level variable, with district-specific values ranging from 0 to 1.

In addition to exploring what factors caused variation in knowledge and application of exemption policies, we included a secondary outcome, asking health workers to identify what proportion of the total population should be considered ultra-poor and therefore be exempted from paying user fees. Five categorical options were provided (1%, 5%, 10%, 20%, 25%).

### Analytical approach

We used univariate descriptive statistics to explore the distribution of the variables of interest in the sample. We ran an ANOVA model with a Sidak multiple-comparison test to identify statistically significant differences across districts for each of the four outcome variables. Given the large number of districts in our sample, we grouped districts that displayed values close to average (i.e., districts whose values on the four outcome variables on average deviated less than ten percentage points in either direction) into one category and compared them against the 10 remaining districts, whose values on average diverged by more than +0.1 or −0.1 from the mean of the four outcome variables.

We ran four random effects models (one for each outcome variable) to identify associations between outcomes of interest and selected sociodemographic and health facility characteristics of health workers. We used random effects models because we wanted to account for potential clustering at the district level, based on the large observed differences in knowledge and application of the exemption mechanisms that emerged from our initial descriptive analysis. All four models accounted for clustering at the district rather than the facility level for two reasons. First, descriptive statistics suggested the presence of district-level effects rather than of facility-level effects. Conceptually, this might be due to the influence of the district health management team as well as to the mobility between facilities within a given district, a phenomenon that is well-known in Burkina Faso [34, 35] and that provides a conceptual grounding for our empirical analytical choice. Second, the number of health workers interviewed in each facility was very small (fewer than three health workers in two-thirds of all facilities), which did not allow for meaningful clustering at the facility level.

Specifically, we ran multilevel models with random coefficients in Stata version 13.1, using the xtlogit command. We expressed our effect estimates as beta coefficients and displayed the standard errors. In the regression equation, Y represented outcome variables, while X represented explanatory variables. In a multilevel model, we assumed that each class (subscript j), here districts, had a different intercept coefficient (β_0 j_) and different slope coefficients ((β_1 j_, β_2 j_,…). Subscript i represented the individual units, here health workers; e _ij_ represented the residual errors. The equation for each of the four multi-level regression models was as follows:

1 $$ {\mathrm{Y}}_{\mathrm{ij}}={\upbeta}_{0\ \mathrm{j}}+{\upbeta}_{1\ \mathrm{j}}{\mathrm{X}}_{1\ \mathrm{ij}}+{\upbeta}_{2\ \mathrm{j}}{\mathrm{X}}_{2\ \mathrm{ij}}+{\mathrm{e}}_{\mathrm{ij}}. $$

## Results

Table 2 provides basic descriptive statistics regarding the explanatory variables in our sample. The sample was equally distributed between men and women, with nearly 90% of all health workers having completed either junior high school (grade 10) or senior high school (grade 13). Over one-fifth of all respondents were AISs, while medical doctors and nurses made up 42% of the sample and midwives and assistant midwives, 36%. Just over one-quarter of all respondents were facility heads. On average, health workers reported having been assigned to a given facility for 3 years and having missed 3 days of work during the preceding month. Nearly 70% of all respondents had undergone a performance evaluation within the past year, but very few had participated in any training. Nearly 90% of all health workers surveyed worked in a first-line facility. Differences in numbers of respondents across districts reflect underlying differences in district size.

**Table 2:** Univariate descriptive statistics

	Mean / %	SD	*N* / *n*	min	max
Outcome indicators					
Knowledge of indigent exemptions	7.09	0.2568	1311	0	1
Application of indigent exemptions (recoded)	5.03	0.2187	1311	0	1
Knowledge of delivery exemptions	25.86	0.4380	1311	0	1
Application of delivery exemptions (recoded)	23.26	0.4227	1311	0	1
Individual characteristics					
Sex	100.00%		1311		
Male	50.19%	0.5002	658	0	1
Female	49.81%	0.5002	653	0	1
Age	34.6	5.3925	1311	20	57
Education	100.00%		1311		
No education or primary	2.75%	0.1635	36	0	1
Junior high school	29.82%	0.4577	391	0	1
Senior high school	58.73%	0.4925	770	0	1
Higher education	8.70%	0.2819	114	0	1
Health worker type	100.00%		1311		
Nurse (IDE, IB, nursing assistant) or medical doctor	42.49%	0.4945	557	0	1
Midwife (midwife/birth attendant/assistant midwife (AA, AB))	35.70%	0.4793	468	0	1
Mobile health worker (AIS)	21.82%	0.4132	286	0	1
Head of health facility	28.38%	0.4510	1311	0	1
Years worked at facility	3.0	2.5925	1311	0	24
Absence in past 30 days	3.2	5.5600	1311	0	30
Performance feedback received during past 12 months	68.96%	0.4629	1311	0	1
Training – emergency obstetric and neonatal care	5.57%	0.2294	1311	0	1
Training – facility (financial) management	1.68%	0.1285	1311	0	1
Influence on decisions (scale from 0 = lowest to 10 = highest)	5.9	3.0557	1311	0	10
Control over facility (scale from 0 = lowest to 10 = highest)	6.8	2.9283	1311	0	10
Facility-level variables					
Environment	100.00%		1311		
Rural	76.74%	0.4227	1006	0	1
Urban	23.26%	0.4227	234	0	1
Type of healthcare facility	100.00%		1311		
Primary	87.80%	0.3275	1151	0	1
Secondary	10.22%	0.3030	134	0	1
Private/other	1.98%	0.1395	26	0	1
Number of clinical staff	3.7	2.2537	1311	1	12
District-level variables					
Share of indigents in district (district-level variable)	22.03%	0.1196	24	0	0.6
District	100.00%		1311		
Boromo	1.60%	0.1256	21	0	1
Nouna	8.62%	0.2808	113	0	1
Solenzo	7.09%	0.2568	93	0	1
Toma	2.67%	0.1613	35	0	1
Manga	2.14%	0.1446	28	0	1
Ouargaye	2.59%	0.1590	34	0	1
Tenkodogo	2.59%	0.1590	34	0	1
Zabre	0.76%	0.0870	10	0	1
Barsalgho	1.30%	0.1132	17	0	1
Kaya	12.20%	0.3275	160	0	1
Kongoussi	6.03%	0.2381	79	0	1
Ziniare	3.81%	0.1916	50	0	1
Doudougou	10.07%	0.3010	132	0	1
Nanoro	1.37%	0.1164	18	0	1
Reo	3.28%	0.1782	43	0	1
Sapouy	3.97%	0.1952	52	0	1
Bousse	3.05%	0.1721	40	0	1
Gourcy	6.18%	0.2409	81	0	1
Ouahigouya	13.42%	0.3410	176	0	1
Yako	3.20%	0.1762	42	0	1
Batie	1.07%	0.1028	14	0	1
Dano	0.84%	0.0913	11	0	1
Diebougou	1.91%	0.1368	25	0	1
Gaoua	0.23%	0.0478	3	0	1
Secondary outcome indicator					
Preference for selection of % of the population to be fully exempted	100,00%		1445		
1%	5.81%	0.2341	84	0	1
5%	24.08%	0.4277	348	0	1
10%	33.08%	0.4707	478	0	1
20%	16.54%	0.3717	239	0	1
25%	20.48%	0.4037	296	0	1

Table 2 also displays health workers’ opinions regarding the percentage of poor people that should be targeted for exemption. Nearly 60% of the health workers indicated a preference for either 5% or 10% as appropriate targeting goals.

Table 3 provides details on the proportions of respondents who knew about the existence of either exemption policy and of those who applied either of them. Both knowledge and application of the indigent exemption for preventive and curative services were extremely low, with only 9.2% of all respondents knowing of its existence and 5% implementing it. Knowledge and application of the delivery exemption were higher, with 27% of all health workers being aware of it and 24.2% applying it. Substantial differences were observed across districts and between the two policies. For instance, only the districts of Nouna and, to a lesser extent, Bousse displayed significantly high levels of knowledge and application for both sets of exemption directives, while other districts, such as Batie, Nanoro, and Diebougou, where knowledge and application of the delivery exemption were the highest, displayed very low levels of knowledge and application of the indigent exemption. In these three districts, as well as in Yako, not a single health worker reported applying the indigent exemption policy.

**Table 3:** Comparison of means of selected districts (percentages of health workers knowing/applying the indigent and delivery directives)

DISTRICTS	(1) Knowledge of indigent exemptions (%)	*N*	(2) Application of indigent exemptions (recoded) (%)	*N*	(3) Knowledge of delivery exemptions (%)	*N*	(4) Application of delivery exemptions (recoded) (%)	*N*
Boromo	7.14	28	3.70	27	53.57*	28	53.57**	28
Nouna	30.30***	132	21.49***	121	50.00***	134	43.28***	134
Manga	6.67	30	3.45	29	3.33	30	3.33	30
Kaya	2.30	174	1.16	173	6.90***	174	3.45***	174
Kongoussi	1.19	84	0.00	83	16.87	83	15.66	83
Nanoro	9.52	21	0.00	19	61.90**	21	61.90**	21
Bousse	26.67**	45	17.07**	41	37.78	45	35.56	45
Gourcy	3.57	84	1.20	83	14.29	84	10.71	84
Batie	4.35	23	0.00	19	73.91***	23	68.18***	22
Diebougou	6.25	32	0.00	41	59.38**	32	59.38***	32
All other districts	8.12	850	4.19	836	25.71	848	23.40	846
Total	9.18	1503	4.98	1472	26.96	1502	24.21	1499

Selected districts with positive and significant relationships with the dependent variables. Districts grouped in “other” were all those whose value deviated from the weighted average of the outcome indicators by no more than one point in either direction. Multiple comparison test applying Sidak correction. Comparison of means of specific district vs. category “all other districts”. Significance levels: * p < 0.05, ** p < 0.01, *** p < 0.001

Table 4 displays the estimates of the four random effects models. The models identified only a handful of statistically significant associations between the health workers’ sociodemographic characteristics, the traits of the facility where they worked, and the four outcomes of interest. AISs were consistently better informed and more likely to apply exemptions, both for indigents and for women at delivery. Compared to other health workers, facility heads were significantly more likely to know (0.922; *p* < 0.01) and apply (2.031; *p* < 0.001) the indigent exemption, but not the delivery exemption. Health workers who perceived themselves as being in control of work at their facility were consistently more likely to know indigents (0.100; *p* < 0.05) and delivery exemptions (0.118; p < 0.001) and significantly more likely to apply the delivery exemption (0.098; p < 0.01). Health workers in districts with higher proportions of very poor people were extremely more likely to know (5.954; p < 0.01) and apply (4.921; p < 0.05) the delivery exemption. The results of all four models confirmed the persistence of a district-level effect, i.e., the presence of district-level variance that could not be explained by the variables included in the model.

**Table 4:** Multilevel logistic regression (Random effects, sub-level: DISTRICT) (coefficients)

	(1) Knowledge of indigent exemptions		(2) Application of indigent exemptions (recoded)		(3) Knowledge of delivery exemptions		(4) Application of delivery exemptions (recoded)	
Sex female (base: male)	−0.048	(0.2897)	−0.096	(0.4238)	0.178	(0.1849)	0.342	(0.1927)
Education (base: Senior high school)								
No education or primary	−0.662	(0.7971)	−0.866	(1.1117)	0.258	(0.4038)	0.352	(0.4069)
Junior high school	0.210	(0.2557)	0.056	(0.3718)	0.563^***^	(0.1648)	0.472^**^	(0.1710)
Higher education	0.0795	(0.3801)	0.707	(0.4665)	−0.517	(0.3084)	−0.225	(0.3122)
Age (centred)	0.0063	(0.0212)	−0.016	(0.0298)	−0.026	(0.0146)	−0.026	(0.0152)
Type of health worker (base: Midwife (midwife/birth attendant/assistant midwife (AA, AB))								
Nurse (IDE, IB, nursing assistant) or medical doctor	0.030	(0.3423)	−0.187	(0.5488)	0.207	(0.2166)	0.316	(0.2283)
Mobile health worker (AIS)	0.436	(0.3535)	1.181^*^	(0.5422)	0.372	(0.2137)	0.564^*^	(0.2215)
Head of health facility	0.922^**^	(0.3271)	2.031^***^	(0.5065)	0.076	(0.2203)	0.180	(0.2293)
Years worked at facility (centred)	−0.010	(0.0452)	0.003	(0.0685)	−0.018	(0.0314)	−0.030	(0.0334)
Absence in past 30 days (centred)	0.008	(0.0178)	0.039	(0.0217)	−0.024	(0.0141)	−0.022	(0.0146)
Performance feedback received during past 12 months	0.262	(0.2441)	0.194	(0.3623)	−0.039	(0.1593)	−0.121	(0.1663)
Training – emergency obstetric and neonatal care	0.026	(0.5235)	−0.991	(1.0983)	−0.050	(0.3274)	−0.417	(0.3655)
Training – facility (financial) management	0.122	(0.6932)	−0.624	(1.1343)	−0.409	(0.5998)	−0.554	(0.6689)
Influence on decisions	−0.059	(0.0378)	−0.057	(0.0551)	0.012	(0.0260)	0.005	(0.0269)
Control over facility	0.100^*^	(0.0463)	−0.000	(0.0645)	0.118^***^	(0.0300)	0.098^**^	(0.0310)
Urban (base: Rural)	0.509	(0.3352)	0.403	(0.5115)	−0.027	(0.2365)	0.117	(0.2459)
Health facility level (base: Primary)								
Secondary	−0.398	(0.5728)	−0.471	(1.0077)	−0.640	(0.3812)	−0.782	(0.4219)
Private/other	0.299	(0.8557)	0.711	(1.1867)	1.040	(0.5336)	0.351	(0.6395)
# of clinical staff	−0.056	(0.0767)	−0.158	(0.1175)	−0.001	(0.0516)	−0.061	(0.0558)
Share of indigents (aggr. by district)	−3.335	(3.1934)	−5.359	(5.6452)	5.954^**^	(1.8221)	4.921^*^	(2.0305)
Constant	−2.993^***^	(0.8719)	−3.459^*^	(1.3780)	−3.416^***^	(0.5658)	−3.113^***^	(0.6109)
lnsig2u	0.142	(0.4145)	1.032^*^	(0.5047)	−0.642	(0.4059)	−0.378	(0.3955)
sigma_u	1.074	(0.2225)	1.675	(0.4228)	0.726	(0.1472)	0.828	(0.1637)
rho	0.259	(0.0797)	0.460	(0.1254)	0.138	(0.0483)	0.172	(0.0564)
Observations	1345		1314		1345		1343	
Wald chi^2^(20)	31.64^*^		43.58^**^		79.55^***^		69.38^***^	
LR ratio test of rho, chibar^2^(01)	72.53^***^		67.76^***^		56.43^***^		68.18^***^	

Standard errors in parentheses. Significance levels: ^*^ *p* < 0.05, ^**^ *p* < 0.01, ^***^ *p* < 0.001

## Discussion

### Persistence of implementation gap impeding equity

The results are relatively disturbing, as they show that very little has changed since the early 2000s. Health workers have little concern for equity issues in West Africa [36] and may not be sufficiently empowered to promote equity in health. This study confirmed our earlier qualitative and quantitative studies, which showed that these user fee exemption policies were not being sufficiently applied [22, 37]. The State decided on several measures to support the worst-off and did so without external funding, which is remarkable and speaks to the high level of political will for equity, especially in the delivery exemption for the indigents [30]. However, political will cannot stop at declarations and the formulation of public policies; measures must be put in place to support their application. This gap was again noted recently in Burkina Faso in the context of a national program to cover indigents using mutual health insurance programs. The program’s content was formulated and funded, but it was never properly implemented, such that potential indigent beneficiaries were once again left out [38]. Implementation science has a long history, yet the lessons it has offered for decades seem to have not yet come to the attention of decision-makers [8, 39]. Even though a causal link between knowledge and its application is not guaranteed, as long as street-level workers do not know the content of policies, they will certainly not be able to apply them. We have shown in previous work that issues of equity and questions relating to exemption policies are not on the health-related curricula in Burkina Faso [40], and they are no longer a topic of discussion in national policy dialogues.

Our study suggests that only a small proportion of health workers appear to know about issues pertaining to targeting and exemption. That suggestion appears to be confirmed by parallel data indicating that less than 2% of all health workers interviewed reported ever having received explicit training on either exemption policy. As such, there is definitely a pressing need for both basic and continuing education on this issue if any action is to be taken to close the obvious implementation gap. Furthermore, the fact that health workers would like to keep the indigent selection coverage to a low level (Table 2 shows that about 30% of health workers indicated less than 5% as an appropriate targeting goal in a country where 9% of the population lives in situations of extreme poverty) confirms prior evidence [41] and may not be due only to lack of training, but also to a lack of concern for equity, as indicated in earlier research [22, 40]. Current payment systems, however, are also likely to be largely responsible for the low application of the exemption policy, given the net gain that health providers make on consultation fees and drug sales. In addition, health workers may be also sincerely worried about the impact of user fees exemptions on health centres’ financial sustainability in a context of cost-recovery schemes and long reimbursement delays. A comparison of the implementation processes for three indigent targeting models in one district of the country showed, in fact, that health workers had little financial incentive to exempt indigents from paying user fees [42]. A pilot project begun in 2015 to exempt indigents from user fees in 12 districts of the country, in which consultations and health worker bonuses are being paid by the World Bank [31], is expected to produce valuable lessons on the effects of strategic purchasing on equity and on the use of services by the worst-off.

### The key roles of context and actors

This quantitative study provides new and very useful knowledge for decision-makers, while also opening up new and important research needs. For example, the amount of variance in the outcome variable that is due to districts cannot be explained by our model. Here is where it is essential to study the role of context, but this is still rarely done in this type of interventional research [43]. Even though the concept of context is still at a stage that is not very “mature” [44], it has been used to understand the heterogeneity of impacts of the delivery subsidy policy in a district in the north of the country [12], as well as why some women in a western district continued to give birth at home [45] despite this policy. It is thus important to undertake qualitative research to understand the observed differences in the knowledge and application of these policies between districts.

At the same time, however, this study was able to show the preponderant role of certain contextual elements in explaining the results. For example, the distribution of respondents in our samples suggests that AISs were often present as healthcare providers in the centres at the time of the survey. Our experience and evidence [12, 27, 46] suggests that it is also very likely that this is the case every day, and therefore that AISs are the ones most in contact with populations and health services users. Workers who are more qualified or have more responsibilities are more often absent and so do not attend training sessions or go to meetings far from their health centre. It is therefore logical that AISs are the ones who are best informed and most concerned about this issue, and that they would report applying the measures to a greater extent. It would certainly be interesting to consider relying more heavily on these health workers, who need to be better trained, to ensure more diligent application of the indigent policies.

Likewise, the leadership role of health centre heads or district chief medical officers, well understood in the country [34] was again highlighted by the results of this study. The directive to exempt indigents was kept very confidential; only those in charge received it. They were supposed to distribute the message to the health centres, but this was not really done, as is often the case in West Africa [47]. Top-down transmission of information in a pyramidal structure is often not effective, as we showed recently in the case of PBF in Burkina Faso [42]. It would be interesting to discuss this hypothesis with the district chief medical officers to understand how information about these directives is distributed, knowing there must still be large differences among districts.

The environment in which health workers live and work may also influence their ideas and views regarding poverty and the needs of surrounding populations. This could explain why those living in districts with higher proportions of indigents were more likely to know and apply the exemption measures for deliveries but not for indigent healthcare. This difference might be due to the fact that the national policy on deliveries was more recent and better organized [30] than the indigent care policy, which remained more confidential [48]. Moreover, we saw that those working in districts where efforts to support indigents were actually implemented (e.g. Nouna [49]) knew more about the exemption measures than did others. The role of ideas in public policy implementation has been widely debated in the scientific literature, both with respect to public policies in general [4] and to policies on user fee exemptions [50]. This study could show that, in fact, there is a link.

### Methodological considerations

The strength of this study lies in the large sample size (1345 health workers on the primary outcomes) and geographic spread (24 districts), as well as in the application of multilevel regression modelling techniques to assess associations between street-level workers’ knowledge and application of exemption policy and socio-demographic and health-facility factors. The large sample size contributes to the external validity of the study, providing reassurance that the results represent the variety of experiences across the entire country. The application of multilevel modelling techniques made it possible to produce a reliable analysis to identify relevant associations with the outcomes of interest.

Nevertheless, we cannot exclude the presence of selection bias, since, as explained in the methods section, the combination of the time available for the survey and the caseload at the facility, allowed us to interview only 80% of all health workers present on the day of the interview. We have no means of assessing if the non-interviewed health workers differed significantly from the interviewed ones. In addition, we have to acknowledge possible social desirability reporting bias, especially in relation to questions on the application of the exemption mechanisms, and that actual behaviour in applying the exemptions may differ from what was declared during the survey. In addition, the lack of district-level explanatory variables (such as district leadership and management, district wealth, existing health interventions, etc.) made it impossible for us to expand the model and explain the district-level variance that persisted across all models. Further studies looking into district-level characteristics are urgently needed to explain the observed geographical variance.

## Conclusion

This study needs to be seen in the context of the new national health financing strategy (2016–2030), which emphasizes the importance of improving the functioning of these exemption measures on the way to achieve universal health coverage. It will be impossible to achieve universal coverage by 2030 without formulating, funding, applying, and evaluating measures specifically targeting the worst-off [16]. Political will, once declared, must be followed by application.

This study showed that there is a significant gap between issuing directives and the actual inadequacy of knowledge and application of these directives in the country’s health facilities. Our results indicate that substantial training of healthcare staff (in addition to strong MoH leadership to ensure the application) is required to make exemption policies effective. Furthermore, transmission of knowledge to health workers within facilities, whether from facility heads or from health workers in charge of the facility, needs to be ensured.

Additionally, it should be noted that thus far, the majority of public health interventions have been focused on improving either the service offer (e.g. quality, training) or accessibility (e.g. financial, geographic). However, the determinants of access to care are numerous, and it is certainly time to mobilize collectively to combine policies that reinforce the quality of services with those that can ensure greater equity, particularly with respect to the worst-off. Formulating exemptions for indigents and improving their social determinants of health to support their access to care health care [24, 51] is one step to achieving greater equity, as long as it can be ensured that those exemptions are actually then implemented and applied in health facilities.

## Additional file

Additional file 1: International Standard Classification of Occupations in Burkina Faso. (DOCX 72 kb)

## Abbreviations

AA: Accoucheuses auxiliaires / Assistant midwife
AB: Accoucheuses brevetées / Midwife
AIS: Agent itinérant de santé / Mobile health worker
CSPS: Centres de santé et de promotion sociale / First-line health centre
IB: Infirmier breveté / Nurse
IDE: Infirmier diplomé d’État / State nurse
PBF: Performance-based financing
WB: World Bank
