# Supplementary material for: Street-level workers’ inadequate knowledge and application of exemption policies in Burkina Faso jeopardize the achievement of universal health coverage: evidence from a cross-sectional survey
Source: Int J Equity Health. 2018 Jan 8;17:5. doi: 10.1186/s12939-017-0717-5 (PMC5759863; doi:10.1186/s12939-017-0717-5)
Supplement: Additional file 1: — International Standard Classification of Occupations in Burkina Faso. (DOCX 72 kb) [file 12939_2017_717_MOESM1_ESM.docx]

**Additional file 1.** International Standard Classification of Occupations in Burkina Faso

| **Major groups, sub-major groups, minor groups, unit groups** |
| --- |
| ***(2) Intellectual and scientific occupations***  (22) Health specialists  (221) Physicians  (2211) Generalist physicians  (2212) Specialist physicians  (222) Nursing personnel and midwives  (2221) Nursing personnel  (2222) Midwives  (226) Specialists in other health occupations  (2269) Health specialists not classified elsewhere |
| ***(3) Intermediate occupations***  (32) Intermediate health occupations  (322) Nursing personnel and midwives (intermediate level)  (3221) Nursing personnel (intermediate level)  (3222) Midwives (intermediate level)  (325) Other intermediate health occupations  (3253) Community health workers  (3259) Intermediate health occupations not classified elsewhere  (323) Practitioners of traditional medicine and of complementary medicine  (3230) Practitioners of traditional medicine and of complementary medicine |

Inspired by: International Standard Classification of Occupations (ISCO-08). Burkina Faso, *United Nations Statistics Division*, 2008, <http://unstats.un.org/unsd/cr/ctryreg/ctrydetail.asp?id=1444>
